# Expression and subcellular localization of cyclin D1 protein in epithelial ovarian tumour cells

**DOI:** 10.1038/sj.bjc.6690826

**Published:** 1999-12

**Authors:** K K Dhar, K Branigan, J Parkes, R E J Howells, P Hand, C Musgrove, R C Strange, A A Fryer, C W E Redman, P R Hoban

**Affiliations:** Centre for Cell and Molecular Medicine, University of Keele School of Postgraduate Medicine, North Staffordshire Hospital, Stoke-on-Trent, UK; Department of Pathology, Stafford District General Hospital, Weston Road, Stafford, UK; Department of Obstetrics and Gynaecology, City General Hospital, Stoke-on-Trent, UK; Department of Histopathology, Central Pathology Laboratories, Stoke-on-Trent, UK

**Keywords:** ovarian cancer, cyclin D1, cytoplasm: nucleus, amplification, clinical outcome

## Abstract

The expression of cyclin D1 protein in tumour sections from 81 patients with epithelial ovarian cancer was analysed using immunohistochemistry. The tumours that overexpressed cyclin D1 in more than 10% of neoplastic cells were considered positive. Thus overexpression of cyclin D1 was observed in 72/81 (89%) of the cases examined. Protein was detected in both the nucleus and the cytoplasm in 24/81 (30%) and localized exclusively in the cytoplasm in 48/81 (59%) of the tumours. Cyclin D1 was overexpressed in both borderline and invasive tumours. There was no association between protein overexpression and tumour stage and differentiation. Furthermore, no correlation between cyclin D1 expression and clinical outcome was observed. However, in tumours overexpressing cyclin D1 (*n* = 72), the proportion displaying exclusively cytoplasmic localization of protein was higher in those with serous compared with non-serous histology (*P* = 0.004, odds ratio 4.8, 95% confidence interval 1.4–19.1). Western analysis using a monoclonal antibody to cyclin D1 identified a 36 kDa protein in homogenates from seven tumours displaying cytoplasmic only and one tumour demonstrating both nuclear and cytoplasmic immunostaining. Using restriction fragment length polymorphism polymerase chain reaction and PCR-multiplex analysis, amplification of the cyclin D1 gene (*CCNDI*) was detected in 1/29 of the tumours demonstrating overexpression of cyclin D1 protein. We conclude that deregulation of *CCND1* expression leading to both cytoplasmic and nuclear protein localization is a frequent event in ovarian cancer and occurs mainly in the absence of gene amplification. © 1999 Cancer Research Campaign


*British Journal of Cancer* (1999) **81**(7), 1174–1181
© 1999 Cancer Research Campaign
Article no. bjoc.1999.0826

### Expression and subcellular localization of cyclin D1 protein in epithelial ovarian tumour cells

**KK Dhar[1,3], K Branigan[1], J Parkes[2], REJ Howells[3], P Hand[4], C Musgrove[4], RC Strange[1], AA Fryer[1], CWE Redman[3] and PR Hoban[1]**

[1]Centre for Cell and Molecular Medicine, University of Keele School of Postgraduate Medicine, North Staffordshire Hospital, Stoke-on-Trent, UK; [2]Department of Pathology, Stafford District General Hospital, Weston Road, Stafford, UK; [3]Department of Obstetrics and Gynaecology, City General Hospital, Stoke-on-Trent, UK; [4]Department of Histopathology, Central Pathology Laboratories, Stoke-on-Trent, UK

**Summary** The expression of cyclin D1 protein in tumour sections from 81 patients with epithelial ovarian cancer was analysed using immunohistochemistry. The tumours that overexpressed cyclin D1 in more than 10% of neoplastic cells were considered positive. Thus overexpression of cyclin D1 was observed in 72/81 (89%) of the cases examined. Protein was detected in both the nucleus and the cytoplasm in 24/81 (30%) and localized exclusively in the cytoplasm in 48/81 (59%) of the tumours. Cyclin D1 was overexpressed in both borderline and invasive tumours. There was no association between protein overexpression and tumour stage and differentiation. Furthermore, no correlation between cyclin D1 expression and clinical outcome was observed. However, in tumours overexpressing cyclin D1 ($n$ = 72), the proportion displaying exclusively cytoplasmic localization of protein was higher in those with serous compared with non-serous histology ($P$ = 0.004, odds ratio 4.8, 95% confidence interval 1.4–19.1). Western analysis using a monoclonal antibody to cyclin D1 identified a 36 kDa protein in homogenates from seven tumours displaying cytoplasmic only and one tumour demonstrating both nuclear and cytoplasmic immunostaining. Using restriction fragment length polymorphism polymerase chain reaction and PCR-multiplex analysis, amplification of the cyclin D1 gene (*CCNDl*) was detected in 1/29 of the tumours demonstrating overexpression of cyclin D1 protein. We conclude that deregulation of *CCND1* expression leading to both cytoplasmic and nuclear protein localization is a frequent event in ovarian cancer and occurs mainly in the absence of gene amplification. © 1999 Cancer Research Campaign

**Keywords:** ovarian cancer; cyclin D1; cytoplasm: nucleus; amplification; clinical outcome

A common target in carcinogenesis is de-regulation of controlled G1-S phase progression of the cell cycle (Palmero and Peters, 1996). Thus during tumourigenesis the genes encoding proteins that regulate progression through G1, frequently undergo genetic alterations. In dividing cells, the cyclin D1 gene (*CCND1*) acts as a growth factor sensor that responds to mitogenic signals to promote cellular proliferation. This is achieved through binding of cyclin D1 protein to specific cyclin-dependent kinases (cdk4 and cdk6). The cdk–cyclin D1 complex catalyses the phosphorylation of the retinoblastoma protein (pRB). In a hyperphosphorylated form pRb is inactivated and cells are released from G1 arrest and progress into S phase (Sherr, 1996).

In many cancers *CCND1* expression is de-regulated leading to overexpression of cyclin D1 protein. This can occur through several mechanisms. For example, in some parathyroid adenomas an inversion involving 11q13 and 11q15 results in *CCND1* expression coming under the control of the parathyroid hormone gene promoter (Motokura et al, 1991). In B-cell malignancies, including centrocytic B-cell lymphomas, *CCND1* expression is de-regulated under the influence of the immunoglobulin heavy chain enhancer as a result of a reciprocal chromosomal translocation at the *BCL1* breakpoint t(11;14)(q13;q32) (de Boer et al, 1993, 1995). Amplification of *CCND1* has been described in other tumours including those of the head and neck, breast, lung, bladder, pancreas and liver (Callender et al, 1994; Gillett et al, 1994; Nishida et al, 1994; Betticher et al, 1996; Bringuier et al, 1996; Gansauge et al, 1997).

Rearrangement of the *CCND1* locus resulting in protein overexpression has been associated with prognosis in a variety of malignancies. In head and neck cancers, *CCND1* amplification, and protein overexpression correlates with poor prognosis (Michalides et al, 1994; Bellacosa et al, 1996). In contrast, cyclin D1 overexpression has been shown to correlate with good prognosis in non-small-cell lung cancer (NSCLC), breast cancer and bladder cancer (Betticher et al, 1996; Bringuier et al, 1996; Gillett et al, 1996). Thus a better understanding of the process of de-regulation of the control of *CCND1* expression should help reveal mechanisms of carcinogenesis and also identify prognostic indicators. Most studies examining the expression of cyclin D1 in tumours have used immunohistochemistry (IHC) with monoclonal antibodies to detect the protein on paraffin-embedded tumour sections. Cyclin D1 has been detected predominantly in the nucleus, though cytoplasmic localization of protein has been described in non-Hodgkin's lymphoma (NHL), NSCLC and some breast carcinomas (Nakamura et al, 1994, Betticher et al, 1996, Gillett et al, 1996).

Ovarian cancer has a very poor prognosis with an average 5-year survival of only 30% (Chang et al, 1994). Most patients present with unresectable, advanced disease and are candidates for chemotherapy. Some of these patients demonstrate markedly

*Received 12 October 1998*
*Revised 23 March 1999*
*Accepted 13 May 1999*

*Correspondence to:* PR Hoban improved survival following chemotherapy, whilst others fail to respond or develop chemoresistance. Clearly, there is a need for a greater understanding of the mechanisms involved in disease pathogenesis to allow development of preventive strategies as well as prognostic markers that could be used in patient management. Recent studies using Western analysis have shown that cyclin D1 is overexpressed in the majority of malignant ovarian tumours but is absent in benign lesions (Barbieri et al, 1997). In this analysis the subcellular localization of cyclin D1 was not determined. IHC studies have shown overexpression of cyclin D1 protein in up to 28% of malignant ovarian tumours, suggesting deregulation of cyclin D1 to be important in a sub-group of patients with the disease (Hung et al, 1996; Worsley et al, 1997). In these works only nuclear immunostaining of cyclin D1 was scored. In a pilot study we have found cytoplasmic localization of cyclin D1 in a high proportion of ovarian tumours (Dhar et al, 1997). Accordingly, we have extended this analysis to examine the frequency and subcellular localization of cyclin D1 in epithelial ovarian cancer from 81 cases. In addition we have investigated the mechanism leading to de-regulation of cyclin D1 expression and determined whether overexpression of the protein is associated with clinical outcome in women with the disease.

### MATERIALS AND METHODS

### Patient characteristics and materials

Eighty-one Northern European Caucasian women with histologically confirmed epithelial ovarian cancer were recruited with Ethics Committee approval and informed consent from the North Staffordshire Hospital. These patients have been described in detail (Howells et al, 1998). Tumours were staged by the criteria of the International Federation of Gynaecologists and Obstetricians (FIGO). Histological type and grade were assigned by one specialist pathologist (CM). Survival is defined as the time between diagnosis and death or the time at which the patient was last seen. Progression-free interval (PFI) is defined as the time interval between diagnosis and the occurrence of an event (death, recurrence, or progression of disease), or the last time the patient was seen. Paraffin-embedded ovarian tumour tissue was also obtained. Solid tumour material was collected at time of surgery, snap-frozen in liquid nitrogen and stored at –70°C. Blood samples were collected in EDTA and stored at –20°C.

### Immunohistochemistry

Immunohistochemistry was carried out using a Shandon sequenza (Shandon Scientific Limited, Cheshire, UK) with a modification of the method described by Betticher et al (1996). Briefly, formalin-fixed paraffin sections were air-dried on 2% 3-aminopropyltriethoxysilane-coated (Sigma, Dorset, UK) slides. After dewaxing in xylene, the sections were treated with freshly prepared 3% hydrogen peroxide in methanol for 5 min in order to block endogenous peroxidase activity and rinsed thoroughly in phosphate-buffered saline (PBS) pH 7.2. The sections were then microwaved for 4 × 5 min in EDTA buffer (pH 8.0). Immunohistochemistry was performed using Vectastatin Universal Elite ABC kit (Vector Laboratories, UK). A mouse monoclonal cyclin D1 antibody (DCS-6; Novocastra, Newcastle Upon-Tyne, UK) was used at 1:25 dilution and sections were incubated for 1 h at room temperature. Detection was performed using freshly prepared diaminobenzene (Sigma) at room temperature for 5 min. Sections were counter-stained with haematoxylin, dehydrated and mounted. Controls were provided by a cyclin D1 positive breast cancer section, negatives by omitting primary antibody and including a normal ovary section. Both nuclear and cytoplasmic staining were recorded. Slides were graded as: negative (0–<10% cells stained), + (>10%–<50% cells stained), ++ (>50%–<75% cells stained) and +++ (>75% cells stained). Staining was interpreted independently by KD and PH.

### Western analysis

Ovarian tissue was disrupted using an Omnigene homogenizer and proteins were extracted using standard procedures (Sambrook et al, 1989). Protein concentrations were estimated using the Bio-Rad Protein assay kit with bovine serum albumin (BSA) as the standard. Tissue homogenates (20 μg protein) were separated on 10% polyacrylamide gels in the presence of sodium dodecyl sulphate (SDS). Proteins were transferred by electroblotting onto polyvinyldifluoride (PVDF) membrane (Amersham, Buckinghamshire, UK). Following transfer, filters were blocked in 1% BSA (Sigma) in PBS-T (PBS containing 0.05% Tween-20) for 1 h at room temperature and subsequently washed twice in PBS-T. Blots were incubated with cyclin D1 monoclonal antibody (clone 105 Transduction Laboratories, Lexington, KY, USA) at 1:250 in PBS-T for 1 h at room temperature. Filters were rinsed three times for 5 min in PBS-T and were then incubated with 1:1000 dilution of horseradish peroxidase-conjugated rabbit anti-mouse IgG secondary antibody (Dako Ltd, Cambridge, UK). Following three 5-min washes in PBS-T, cyclin D1 protein was visualized using the super signal substrate (Pierce, Chester, UK). Molecular weight markers (molecular weight standard mixture 29–205 kDa) and glyceraldehyde 3-phosphate dehydrogenase (36 kDa) were used as references for protein size (Sigma). Filters were stripped following the manufacturer's recommendations (Hybond-P, PVDF membrane, Amersham) and re-probed with a monoclonal antibody to vinculin (Sigma) to assess protein loading.

### DNA extraction

DNA was extracted from paraffin sections (5 μm) using a genomic DNA extraction kit (Scotlab, Strathclyde, UK). High molecular weight DNA was extracted from frozen ovarian tissue and peripheral blood using a phenol–chloroform method as described by Sambrook et al (1989).

### PCR analysis of *CCND1*

Imbalances within *CCND1* were assessed by restriction fragment length polymorphism polymerase chain reaction (RFLP-PCR) analysis using polymorphisms in exon 4 and the 3′ UTR region of the gene as described by Betticher et al (1995, 1996). Briefly, PCR products were digested with restriction enzymes to reveal the polymorphic alleles; *Hae*III (New England Biolabs, Hertfordshire, UK) for the 3′UTR polymorphism and *ScrF*I (New England Biolabs) for the exon 4 polymorphism. Digests were visualized on 3% agarose gels stained with ethidium bromide. Multiplex PCR was a modification of the method previously described (Betticher et al, 1996). The *CCND1* gene (chromosome 11q13) was compared to the adenosine deaminase (*ADA*) gene (chromosome 20q12–13). The following primers were used to amplify regions of

© 1999 Cancer Research Campaign 

**Table 1** Cyclin D1 expression and localization in ovarian cancers

| | Cyclin D1 level of staining | | | | |
|---|---|---|---|---|---|
| | 0 | + | ++ | +++ | Total |
| Negative | 9 (11.1) | 0 (0) | 0 (0) | 0 (0) | 9 (11.1) |
| Positive | | | | | |
| Nuclear[a] | 0 (0) | 1 (1.2) | 2 (2.5) | 4 (4.9) | 7 (8.6) |
| Nuclear/cytoplasmic[b] | 0 (0) | 3 (3.7) | 10 (12.3) | 4 (4.9) | 17 (21) |
| Cytoplasmic only | 0 (0) | 24 (29.6) | 12 (14.8) | 12 (14.8) | 48 (59.2) |
| Total | 9 (11.1) | 28 (34.6) | 24 (29.6) | 20 (24.7) | 81 (100) |

Numbers in parentheses are %. [a]Predominantly nuclear staining with some diffuse cytoplasmic localization. [b]Simultaneous nuclear and cytoplasmic immunostaining. Stained sections were graded as negative (0–<10% cells stained), + (>10%–<50% cells stained), ++ (>50%–<75% cells stained) and +++ (>75% cells stained).

the genes; *CCND1*; forward, 5′ATATTCCGTAGGTAGATGTG-TAAC3′ and reverse, 5′TGTCACTATTTCGTCTTCTC3′. ADA; forward, 5′GCGGGTGAACGTCAATGTGTT3′, and reverse, 5′GTGTCCAGGGTGGACTTGAAG3′. Multiplex PCR was performed in 100-μl volumes containing 1 × *Taq* polymerase buffer (Promega UK Ltd, Southampton, UK), a total of 100 μM dNTPs, 0.5 μg of each *CCND1* primer and 0.05 μg of each *ADA* primer, 0.5 μg of genomic DNA and 2 U *Taq* polymerase (Promega). Reactions were cycled 28 times on an automated PCR machine. Products were visualized on 3% agarose gels stained with ethidium bromide. *CCND1* allelic imbalance/gene amplification was determined visually by comparison to normal control DNA samples and the A431 cell line which displays a five-fold level of amplification of *CCND1* (Kurzrock et al, 1995).

### Statistical analysis

Statistical analysis was performed using Stata, version 5 (Stata Corporation, Texas). Pearson-$\chi^2$ tests were used to identify differences between staining patterns and the clinico-pathological parameters. A Cox's proportional hazard regression model was used in the analysis of the effects of cyclin D1 staining on PFI and survival using univariate analysis. Multivariate analysis was used to correct for any potential confounding factors (e.g. age). Kaplan–Meier curves were generated for graphical representation of survival. A probability level of 5% was considered statistically significant.

### RESULTS

### IHC analysis of cyclin D1 expression and localization

Overexpression of cyclin D1 protein was detected in 72/81 (89%) of the epithelial ovarian cancers examined. The level of overexpression was variable between tumours and was graded accordingly (Table 1). Cyclin D1 was detected predominantly in the nuclei of tumour cells in 7/81 (9%) of the cases, and simultaneously in the nucleus and cytoplasm of tumours in 17/81 (21%). Thus 24/81 (30%) of the tumours examined displayed nuclear expression of cyclin D1. However, in 48/81 (59%) of the tumours examined expression was restricted to the cytoplasm of the tumour cells. Representative examples of simultaneous nuclear and cytoplasmic (Figure 1 A, B) and exclusively cytoplasmic staining (Figure 1 C, D) are shown. Paraffin sections obtained from normal ovarian tissue did not demonstrate cyclin D1 immunostaining (Figure 1 E).

### Western analysis of cyclin D1 expression

To investigate the specificity of the observed cytoplasmic staining for cyclin D1, we performed Western analysis and determined the size of the cross-reacting protein. Western blotting for cyclin D1 expression was performed on proteins extracted from 11 malignant tumours and two benign cysts snap-frozen at surgery. The monoclonal antibody clone 105 (Transduction Laboratories) was used to detect cyclin D1. A 36 kDa cyclin D1 protein was detected in 7/7 tumours displaying exclusively cytoplasmic immunostaining of cyclin D1 and 1/1 tumour displaying simultaneous nuclear with cytoplasmic immunostaining. Cyclin D1 was not detected in 3/3 malignant tumours negative for immunostaining (data not shown) and 2/2 benign ovarian cysts. Representative examples of cyclin D1 expression detected by Western blotting in malignant tumours displaying nuclear and cytoplasmic immunostaining, and exclusively cytoplasmic immunostaining are shown in Figure 2. The level of cyclin D1 expression varied between tumours when compared to the intensity of the vinculin blots.

### Analysis of *CCND1* amplification in tumours overexpressing cyclin D1 protein

The mechanism leading to de-regulation of cyclin D1 protein expression in ovarian tumours was investigated. Tissue from 33 tumours displaying overexpression of cyclin D1 protein was available for DNA and was assessed for amplification of *CCND1*. Using multiplex and/or RFLP-PCR techniques on the same sample, information was available on *CCND1* amplification in 29 of the tumours. Amplification of *CCND1* was detected in 1/29 of these tumours. Amplification was assessed qualitatively by comparison of the intensity of ethidium bromide staining of PCR products in tumours with normal controls and the A431 vulval carcinoma cell line (Figure 3 A, B) which has a reported fivefold amplification of *CCND1* (Kurzrock et al, 1995). A representation of allele imbalance detected by RFLP-PCR analysis and amplification of *CCND1* detected by multiplex PCR is shown (Figure 3 A,B). Tumour 323 displayed allele imbalance for *CCND1 Hae*III (Figure 3A, lane 6). This was confirmed as *CCND1* amplification by multiplex PCR (Figure 3B, lane 3). However 5/26 tumours informative for either or both of the *CCND1* polymorphisms displayed allele imbalance in the absence of multiplex-PCR imbalances. For example, tumour 51 displayed imbalance of a *CCND1* allele using RFLP-PCR for the *CCND1 Hae*III and *ScrF*I polymorphisms (Figure 3A, lanes3 & 4 and 9 & 10), but displayed no imbalance with multiplexPCR (Figure 3B, lane 2). These tumours

 © 1999 Cancer Research Campaign

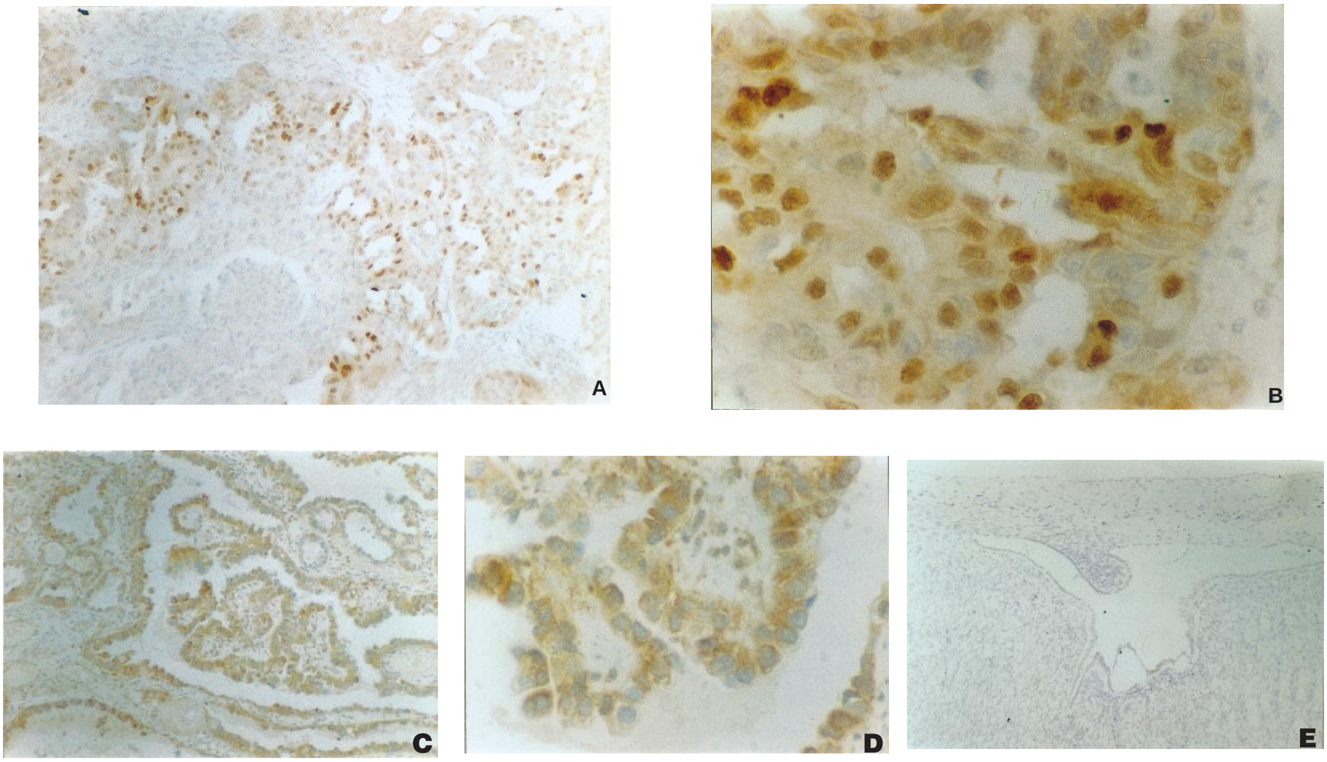

**Figure 1** Immunohistochemical analysis of cyclin D1 expression in epithelial ovarian cancers. Simultaneous nuclear and cytoplasmic localization of cyclin D1 visualized under (**A**) low (×10 objective) and (**B**) high (×40 objective) magnification. Cytoplasmic localization of cyclin D1 visualized under (**C**) low and (**D**) high magnification. Normal ovarian tissue stained for cyclin D1 expression under low magnification (**E**)

were interpreted as displaying allele imbalance at the *CCND1* locus, which may be as a result of LOH of a *CCND1* allele.

### Association of IHC staining of cyclin D1 with tumour characteristics

To investigate whether cyclin D1 protein overexpression and subcellular localization was associated with prognosis, the IHC data was compared with tumour and patient characteristics. Cytoplasmic and nuclear expression of cyclin D1 protein was detected in both borderline and invasive tumours. No significant difference was found in the number of tumours staining positive for cyclin D1 or the subcellular localization of the protein when compared with tumour differentiation and FIGO stage (Table 2). However, a significant difference ($P$ = 0.0137) in the distribution of immunostaining for cyclin D1 was observed when compared with tumour histology (Table 2). In addition, within tumours expressing cyclin D1 ($n$ = 72), cytoplasmic localization occurred in a higher proportion of serous compared with non-serous tumours (Fisher exact test; $P$ = 0.004, OR 4.84, 95% CI 1.42–19.12).

### Association of IHC staining of cyclin D1 with clinical outcome

We compared the expression of cyclin D1 with clinical outcome in 79 patients in whom clinical follow-up data was available (Howells et al, 1998). Using Cox's proportional hazards model we found no significant correlation between expression and subcellular localization of cyclin D1 when compared with progression-free interval (PFI) and overall survival (Figure 4). We also compared the level of expression (neg. & + vs ++ & +++) with PFI and overall survival. The median PFI (23 months vs 23.2 months) and median survival (35 months vs 40.4 months) within these two groups were not significantly different (data not shown).

### DISCUSSION

We have used IHC to detect cyclin D1 overexpression in 81 epithelial ovarian cancers. We found a large proportion of the tumour studied demonstrated overexpression of cyclin D1 protein. Expression was not detected in histologically normal ovarian tissue or benign ovarian cysts. In over half of the tumours expressing cyclin D1, the protein was localized exclusively in the cytoplasm of the cells. Overexpression of cyclin D1 as determined by IHC, has previously been described in ovarian tumours (Hung et al, 1996; Worsley et al, 1997). Hung et al reported overexpression in 28% of cases examined using a polyclonal cyclin D1 antibody whilst Worsley et al detected overexpression of cyclin D1 in up to 26% of tumours using a mouse monoclonal antibody (mAb). The lower number of cases overexpressing cyclin D1 reported in these earlier studies may reflect that only nuclear expression of cyclin D1 was scored. In our series, nuclear expression of cyclin D1 occurs in 30% of ovarian tumours which compares favourably with previous reports.

*© 1999 Cancer Research Campaign* 

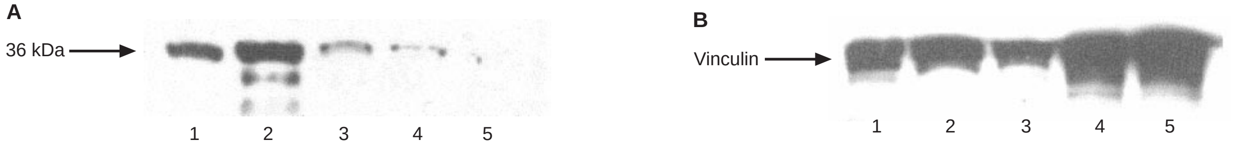

**Figure 2** Expression of cyclin D1 (36 kDa) protein in epithelial ovarian tumours. Lysates from malignant tumours displaying cytoplasmic (lanes 1, 3 & 4) and nuclear/cytoplasmic (lane 2) expression of cyclin D1 (as determined by IHC) are shown. Lane 5 contains lysate extracted from a benign ovarian cyst. Membranes were probed with: (**A**) monoclonal antibody for cyclin D1, the position of the 36 kDa protein is shown left; (**B**) levels of protein loading were visualized by stripping the membrane and re-probing with a monoclonal antibody for vinculin

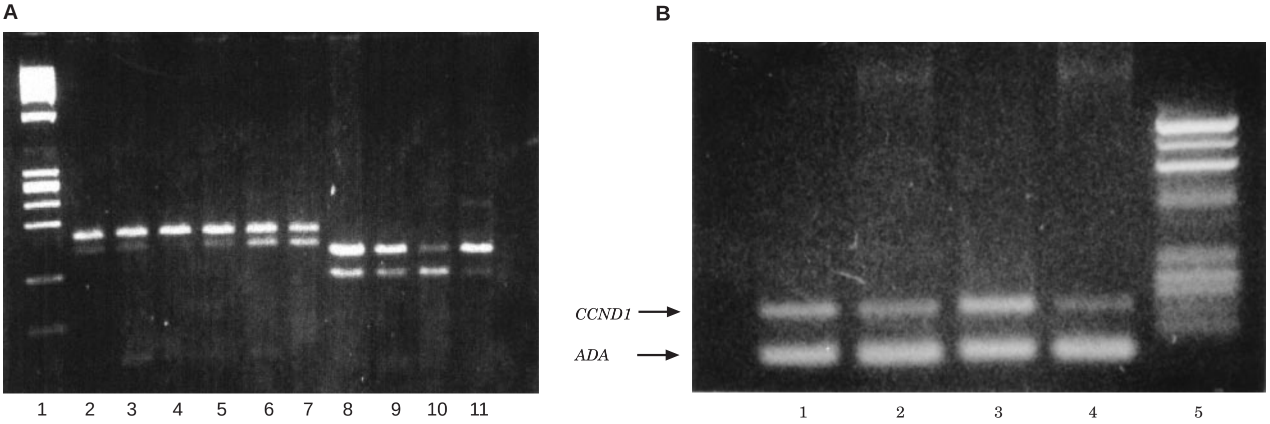

**Figure 3** Analysis of *CCND1* amplification in ovarian tumours. (**A**) RFLP-PCR analysis of *CCND1*; PCR products were digested with *ScrF*I (lanes 2–7) or *Hae*III (lanes 8–11) and separated on 3% agarose gels stained with ethidium bromide. Lane 1; ØX 174 *Hae*III digest, lane 2; normal lymphocytes, lane 3; lymphocytes patient 51, lane 4; tumour patient 51, lane 5; normal lymphocytes patient 323, lane 6; tumour patient 323, lane 7; A431. Lane 8; normal lymphocytes, lane 9; normal lymphocytes patient 51, lane 10; tumour patient 51, lane 11; A431. (**B**) Multiplex PCR analysis of ovarian tumours to determine amplification of *CCND1*. PCR products for *CCND1* and *ADA*, shown left were separated on 3% agarose gels. Lane 1; A431 DNA, lane 2; tumour 51, lane 3; tumour 323, lane 4; normal lymphocytes, lane 5; ØX 174 *Hae*III digest

**Table 2** Association of cyclin D1 expression with ovarian tumour characteristics

| | Cyclin D1 immunostaining | | |
|---|---|---|---|
| | **Nuclear** *n* **= 24 (29.6)** | **Cytoplasmic** *n* **= 48 (59.2)** | **Negative** *n* **= 9 (11.1)** |
| Age at diagnosis (years) | | | |
| Range | 36–74 | 28–90 | 46–80 |
| Median | 59.5 | 61.5 | 74 |
| Tumour differentiation | | | |
| Borderline | 2 (25.0) | 5 (62.5) | 1 (12.5) |
| Well | 4 (57.1) | 3 (42.9) | 0 (–) |
| Moderate | 10 (33.3) | 17 (56.7) | 3 (10) |
| Poor | 5 (28.8) | 15 (62.5) | 4 (16.7) |
| Not mentioned | 3 (25.0) | 8 (66.7) | 1 (8.3) |
| FIGO stage | | | |
| I | 11 (42.3) | 14 (53.9) | 1 (3.8) |
| II | 2 (22.2) | 6 (66.7) | 1 (11.1) |
| III | 9 (22.5) | 24 (60.0) | 7 (17.5) |
| IV | 2 (33.3) | 4 (66.7) | 0 (–) |
| Histology | | | |
| Endometroid | 7 (50) | 6 (42.9) | 1 (7.1) |
| Serous[a] | 5 (13.5) | 27 (73.0) | 5 (13.5) |
| Clear cell | 4 (36.3) | 6 (54.6) | 1 (9.1) |
| Mucinous | 6 (46.2) | 7 (53.8) | 0 (–) |
| Other/Mixed | 2 (33.3) | 2 (33.3) | 2 (33.3) |

Figures in parenthesis are %. [a]The distribution of immunostaining in serous tumours was significantly different compared with other histological types. (Fisher exact test, $P = 0.0137$, $\chi^2_2 = 8.44$).

 © 1999 Cancer Research Campaign

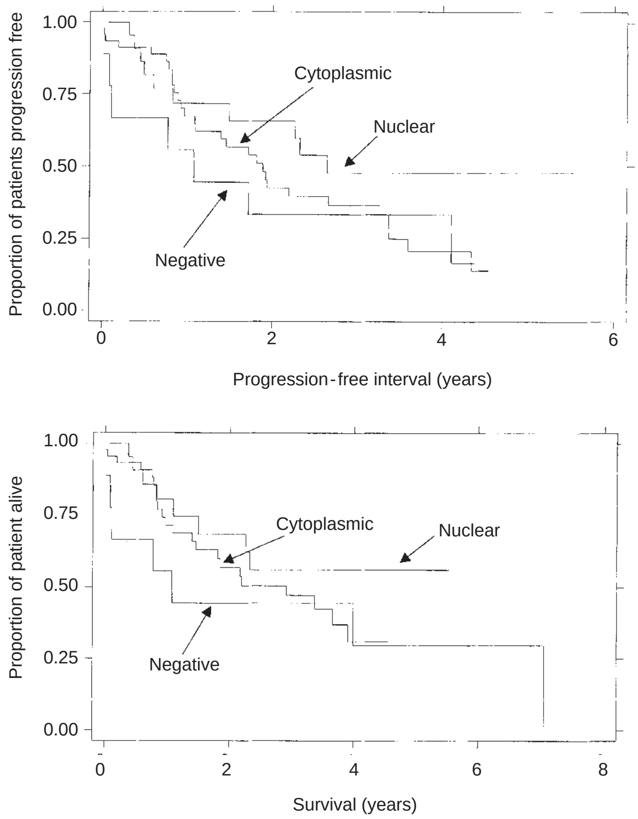

**Figure 4** Kaplan–Meier survival plots showing the association between cyclin D1 expression and (**A**) progression-free interval and (**B**) survival, in patients with epithelial ovarian cancer

Recently using Western analysis, cyclin D1 protein was detected in 90% of the malignant ovarian tumours examined (Barbieri et al, 1997). The high level of cyclin D1 overexpression in this study compared with previous findings (Hung et al, 1996; Worsley et al, 1997) may reflect differences in sensitivity of detection of Western blotting compared with IHC. However, Barbieri et al did not determine subcellular localization of the protein, which prompted us to consider the significance of cytoplasmic staining for cyclin D1. To investigate the specificity of cytoplasmic immunostaining of cyclin D1, we used Western analysis of extracted proteins from tumours demonstrating overexpression of cyclin D1. We used a different cyclin D1 mAb for the Western analysis (clone 105) than that used for IHC (clone DCS-6). Both antibodies identify a 36 kDa protein in Western analysis of a panel of cell lines (data not shown), although in our hands DCS-6 is not as specific as clone 105 in Western blots on ovarian tumour material. Furthermore, clone 105 was not suitable for IHC. Analysis of tumours displaying exclusively cytoplasmic overexpression of cyclin D1 and a tumour displaying simultaneous nuclear and cytoplasmic expression revealed a 36 kDa band with clone 105. We were unable to detect expression of cyclin D1 by Western analysis in three tumours negative by IHC, as well as benign ovarian cysts. Together the IHC and Western data consolidate our findings of frequent cytoplasmic overexpression of cyclin D1 in our cohort of tumours. It is possible, however, that the two antibodies may recognize different cyclin D1 epitopes and further may hybridize to other cyclin D1 protein variants. It has been shown that *CCND1* mRNA is alternatively spliced (Betticher et al, 1995) and that DCS-6 recognizes both proteins encoded from the alternate transcripts in vitro expression studies (Sawa et al, 1998). Future studies including immunoprecipitation experiments are needed to further characterize cytoplasmic cyclin D1 protein in ovarian tumours.

In many cancers overexpression of cyclin D1 protein is associated with tumour characteristics and clinical outcome. The localization of the cyclin D1 protein may have implications for the diagnosis and prognosis in cancer. In NHL localization of cyclin D1 has been implicated in patient prognosis (Nakamura et al, 1994). Thus in mantel cell lymphomas (MCL), generally associated with poor prognosis, frequent chromosomal translocation t(11;14)(q13;q32) leads to strong nuclear overexpression of cyclin D1 protein and distinguishes the malignancy from other types of NHL (Nakamura et al, 1994; de Boer et al, 1995). Nakumura et al also describe a pattern of cytoplasmic immunoreactivity in diffuse B-cell and follicular B-cell NHL which are prognostically different to MCL. In ovarian cancers, we found no correlation between overexpression and localization of cyclin D1 protein with tumour stage or differention. However, cytoplasmic localization of cyclin D1 protein occurred more frequently in serous compared with non-serous tumours. A correlation between nuclear expression of cyclin D1 and well-differentiated and borderline malignancy in a series of 43 ovarian tumours has been described (Worsley et al, 1997). The differences of our findings may reflect the larger number of tumours studied. We did not find any significant associations between cyclin D1 overexpression and subcellular localization with PFI and overall survival of patients. It has been shown that overexpression of cyclin D1 mRNA in epithelial ovarian cancers does not correlate with clinical outcome (Masciullo et al, 1997); however, to date no study has examined expression of cyclin D1 protein with clinical outcome.

The mechanism of cyclin D1 protein overexpression in ovarian cancer is unclear. Amplification of *CCND1* in ovarian tumours is infrequent (Courjal et al, 1996; Masciullo et al, 1997); however, in these studies cyclin D1 protein levels were not measured. We examined *CCND1* amplification in tumours overexpressing cyclin D1 protein as determined by IHC; 28/29 of these tumours did not demonstrate amplification of *CCND1*. However, 5/24 of the tumours displayed allele imbalance of *CCND1* using RFLP-PCR, but an absence of imbalance of *CCND1* using PCR multiplex analysis. These findings may result from LOH at the *CCND1* locus in these tumour cells. High levels of LOH at 11q have been previously described in epithelial ovarian cancers though there is no evidence to suggest that *CCND1* is the target of the LOH in this region (Foulkes et al, 1993; Gabra et al, 1996). Overexpression of cyclin D1 in the absence of gene amplification has been reported in NSCLC and breast cancer (Gillett et al, 1994; Betticher et al, 1996). Clearly mechanisms other than *CCND1* amplification may lead to overexpression of cyclin D1 protein. To date no in vivo abnormalities to *CCND1* have been described that influence intracellular localization of the protein. In vitro mutations to *CCND1* have been shown to influence the localization of cyclin D1–cdk4 protein complexes to the cytoplasm in mouse cells (Diehl and Sherr, 1997). In this study, nuclear localization could be achieved through overexpression of the cdk inhibitor p21. It has been suggested that cdk inhibitors including p21 may have additional roles as adaptor proteins involved in the assembly and intracellular

© 1999 Cancer Research Campaign 

localization of kinase complexes (LaBaer et al, 1997). No mutations in *CCND1* coding sequences were found in NSCLC cells displaying cytoplasmic only expression (Betticher et al, 1995). It is possible however that localization of cyclin D1 to the cytoplasm in ovarian tumours results from mutations to other genes encoding proteins with which it complexes.

Understanding the function of cytoplasmic cyclin D1 in ovarian tumours may be important clinically. In the nucleus cyclin D1 facilitates cell cycle progression. Thus overexpression of cyclin D1 protein in vitro is associated with increased proliferation (Quelle et al, 1993; Musgrove et al, 1994). However, paradoxically, cyclin D1 overexpression has also been associated with apoptosis (Kranenburg et al, 1996; Kotelnikov et al, 1997) and cellular senescence (Dulic et al, 1993; Lucibello et al, 1993). The function of cytoplasmic cyclin D1 described in this study has not been addressed. However, it would be interesting to investigate the expression of cdk4 or the status of pRB in these tumours. In addition a cytoplasmic substrate for cdk4 and cdk6 has been described (Kwon et al, 1995). Furthermore, although predominantly nuclear during the G1 phase of the cell cycle, cyclin D1 and D2 in dividing U-2-OS cells demonstrate cytoplasmic localization at G1/S transition (Lukas et al, 1994).

In conclusion, overexpression of cyclin D1 leading to cytoplasmic and nuclear protein localization is a frequent event in ovarian cancer occurring mainly in the absence of *CCND1* amplification. A better knowledge of the mechanisms involved in deregulation of cyclin D1 expression and the biological consequence of this, may ultimately lead to the development of improved strategies for the treatment of ovarian cancer.

### ACKNOWLEDGEMENTS

We would like to thank the West Midlands NHS Executive, Locally Organised Research Scheme for supporting this work.

### REFERENCES

Barbieri F, Cagnoli M, Ragni N, Pedulla F, Foglia G and Alama A (1997) Expression of cyclin D1 correlates with malignancy in human ovarian tumours. *Br J Cancer* **75**: 1263–1268

Bellacosa A, Almadori G, Cavallo S, Cadoni G, Galli J, Ferrandina G, Scambia G and Neri G (1996) Cyclin D1 gene amplification in human laryngeal squamous cell carcinomas: prognostic significance and clinical implications. *Clin Cancer Res* **2**: 175–180

Betticher DC, Heighway J, Haselton PS, Altermatt HJ, Ryder WDJ, Cerny T and Thatcher N (1996) Prognostic significance of *CCND1* (cyclin D1) overexpression in primary resected non-small-cell lung cancer. *Br J Cancer* **73**: 294–300

Betticher DC, Thatcher N, Altermatt HJ, Hoban P, Ryder WDJ and Heighway J (1995) Alternate splicing produces a novel cyclin D1 transcript. *Oncogene* **11**: 1005–1011

Bringuier PP, Tamimi YE, Schuuring E and Schalken J (1996) Expression of cyclin D1 and EMS1 in bladder tumours: relationship with chromosome 11q13 amplification. *Oncogene* **12**: 1747–1753

Callender T, El-Naggar AK, Lee MS, Frankenthaler R, Luna MA and Batsakis JG (1994) PRAD 1 (CCND1)/Cyclin D1 oncogene amplification in head and neck squamous cell carcinoma. *Cancer* **74**: 152–158

Chang J, Bridgewater J, Gore M, Fischer C, Schofield J, A'Hern R, Ponder B, Jacobs I, McKeage M, Kelland L and Harap K (1994) Non surgical aspects of ovarian cancer. *Lancet* **343**: 335–340

Courjal F, Louason G, Speiser P, Katsaros D, Zeillinger R and Theillet C (1996) Cyclin gene amplification and overexpression in breast and ovarian cancers: evidence for the selection of cyclin D1 in breast and cyclin E in ovarian tumours. *Int J Cancer* **69**: 247–253

de Boer CJ, Loyson S, Kluin PM, Kluin-Nelemans HC, Schruuring E and van Krieken JH (1993) Multiple breakpoints with the *bcl*-1 locus in B-cell lymphoma: rearrangements of the cyclin D1 gene. *Cancer Res* **53**: 4148–4152

de Boer CJ, Schuuring E, Dreef E, Peters G, Bartek J, Kluin PM and van Krieken JH (1995) Cyclin D1 protein analysis in the diagnosis of matel cell lymphoma. *Blood* **86**: 2715–2723

Dhar KK, Branigan K, Hand P, Howells R, Redman C, Strange R, Heighway J and Hoban P (1997) Analysis of Cyclin D1 amplification and overexpression in ovarian cancer. *Acta Obstet Gynaecol Scand* **76**: 19

Diehl JA and Sherr CJ (1997) A dominant-negative mutant prevents nuclear import of cyclin dependent kinase 4 (CDK4) and its phosphorylation by CDK-activating kinase. *Mol Cell Biol* **17**: 7362–7364

Dulic V, Drullinger LF, Lees E, Reed SI and Stein GH (1993) Altered regulation of G1 cyclins in senescent human diploid fibroblasts: accumulation of inactive cyclin E-Cdk2 and cyclin D1-Cdk2 complexes. *Proc Natl Acad Sci USA* **90**: 11034–11038

Foulkes WD, Campbell IG, Stamp G and Trowsdale J (1993) Loss of heterozygosity and amplification on chromosome 11q in human ovarian cancer. *Br J Cancer* **67**: 268–273

Gabra H, Watson JEV, Taylor KJ, Mackay J, Leonard RC, Steel CM, Porteous DJ and Smyth JF (1996) Definition and refinement of loss of heterozygosity at 11q23.3–q24.3 in epithelial ovarian cancer associated with poor prognosis. *Cancer Res* **56**: 950–954

Gansauge S, Gansauge F, Ramadani M, Stobbe H, Rau B, Harada N and Beger H (1997) Overexpression of cyclin D1 in human pancreatic carcinoma is associated with poor prognosis. *Cancer Res* **57**: 1634–1637

Gillett C, Fantl V, Smith R, Fisher C, Bartek J, Dickson C, Barnes D and Peters G (1994) Amplification and overexpression of cyclin D1 in breast cancer detected by immunohistochemical staining. *Cancer Res* **54**: 1812–1817

Gillett C, Smith P, Gregory W, Richards M, Millis R, Peters G and Barnes D (1996) Cyclin D1 and prognosis in human breast cancer. *Int J Cancer* **69**: 92–99

Howells REJ, Redman CWE, Dhar KK, Sarhanis P, Musgrove C, Jones PW, Alldersea J, Fryer AA, Hoban P and Strange RC (1998) Association of glutathione S-transferase GSTM1 and GSTT1 null genotypes with clinical outcome in epithelial ovarian cancer. *Clin Cancer Res* **4**: 2439–2445

Hung W-C, Chai C-Y, Huang J-S and Chuang L-Y (1996) Expression of cyclin D1 and c-Ki-*ras* gene in human epithelial ovarian tumours. *Hum Pathol* **27**: 1324–1328

Kotelnikov VM, Coon JS, Mundle S, Kelanic S, LaFollette S, Taylor IVS, Hutchinson J, Panje W, Caldarelli DD and Preisler HD (1997) Cyclin D1 expression in squamous cell carcinomas of the head and neck and in oral mucosa in relation to proliferation and apoptosis. *Clin Cancer Res* **3**: 95–101

Kranenburg O, van der Eb AJ and Zantema A (1996) Cyclin D1 is an essential mediator of apoptotic neuronal cell death. *EMBO J* **15**: 46–54

Kurzrock R, Ku S and Talpaz M (1995) Abnormalities in the *PRAD1* (*Cyclin D1*/ *BCL-1*) oncogene are frequent in cervical and vulval squamous cell carcinoma cell lines. *Cancer* **75**: 584–590

Kwon TK, Buchholz MA, Gabrielson EW and Nordin AA (1995) A novel cytoplasmic substrate for cdk4 and cdk6 in normal and malignant epithelial derived cells. *Oncogene* **11**: 2077–2083

La Baer J, Garrett MD, Stevenson LF, Slingerland JM, Sandhu C, Chou HS, Fattaey A and Harlow E (1997) New functional activities for the p21 family of CDK inhibitors. *Genes Dev* **11**: 847–862

Lukas J, Bartkova J, Welcker M, Peterson O, Peters G, Strauss M and Bartek J (1995) Cyclin D2 is a moderately oscillating nucleoprotein required for G1 phase progression in specific cell types. *Oncogene* **10**: 2125–2134

Lucibello FC, Sewing A, Brusselbach S, Burger C and Muller R (1993) Deregulation of cyclins D1 and E and suppression of cdk2 and cdk4 in senescent human fibroblasts. *J Cell Sci* **105**: 123–133

Masciullo V, Scambia G, Marone M, Giannitelli C, Ferrandina G, Bellacosa A, Bendetti Panici P and Mancus OS (1997) Altered expression of cyclin D1 and *CDK4* genes in ovarian carcinomas. *Int J Cancer* **74**: 390–395

Michalides R, van Veelen N, Hart A, Loftus B, Wientjens E and Balm A (1995) Overexpression of cyclin D1 correlates with recurrence in a group of forty-seven operable squamous cell carcinomas of the head and neck. *Cancer Res* **55**: 975–978

Motokura T, Bloom T, Kim HG, Juppner H, Ruderman JV, Kronenberg HM and Arnold A (1991) A novel cyclin encoded by a *bcl*-1 linked candidate oncogene. *Nature* **350**: 512–515

Musgrove EA, Lee CS, Buckley MF and Sutherland RL (1994) Cyclin D1 induction in breast cancer cells shorten G1 and is sufficient for cells arrested in G1 to complete the cell cycle. *Proc Natl Acad Sci USA* **91**: 8022–8026

Nakamura S, Seto M, Banno S, Suzuki S, Koshikawa T, Kitoh K, Kagami Y, Ogura M, Yatabe Y, Kojima M, Motoori T, Takahashi T, Ueda R and Suchi T (1994)

 © 1999 Cancer Research Campaign

Immunohistochemical analysis of cyclin D1 protein in hematopoietic neoplasms with special reference to mantel cell lymphoma *Jpn J Cancer Res* **85**: 1270–1279
Nishida N, Fukuda Y, Komeda T, Kita R, Sando T, Furukawa M, Amenomori M, Shibagaki I, Nakao K, Ikenaga M and Ishizaki K (1994) Amplification and overexpression of the cyclin D1 gene in aggressive human hepatocellular carcinoma. *Cancer Res* **54**: 3107–3110
Palmero I and Peters G (1996) Perturbation of cell cycle regulators in human cancer. *Cancer Surveys* **27**: 351–367
Quelle DE, Ashmun RA, Shurtleff SA, Kato JY, Bar-Sagi D, Roussel MF and Sherr CJ (1993) Overexpression of mouse D-type cyclins accelerates G1 phase in rodent fibroblasts. *Genes Dev* **7**: 1559–1571
Sambrook J, Fritsch EF and Maniatis T (1989) *Molecular Cloning: A Laboratory* Manual, 2nd edn. Cold Spring Harbor Laboratory Press: Cold Spring Harbor NY, USA
Sawa H, Ohshima TA, Ukita H, Murakami H, Chiba Y, Kamanda H, Hara M and Saito I (1998) Alternatively spliced forms of cyclin D1 modulate entry into the cell cycle in an inverse manner. *Oncogene* **16**: 1701–1712
Sherr CJ (1996) Cancer cell cycles. *Science* **274**: 1672–1677
Worsley S, Ponder BAJ and Davies BR (1997) Overexpression of cyclin D1 in epithelial ovarian cancers. *Gynecol Oncol* **64**: 189–195

*© 1999 Cancer Research Campaign*
